# First Experience Using a Novel Variable Loop Catheter for Mapping and Pulsed Field Ablation of Atrial Fibrillation

**DOI:** 10.1111/pace.15177

**Published:** 2025-03-28

**Authors:** Thomas Fink, Vanessa Sciacca, Kevin Bannmann, Maximilian Moersdorf, Sebastian Beyer, Alessandro Parlato, Denise Guckel, Mustapha El Hamriti, Moneeb Khalaph, Martin Braun, Maxim Didenko, Guram Imnadze, Dominik Linz, Kevin Vernooy, Philipp Sommer, Christian Sohns

**Affiliations:** ^1^ Clinic for Electrophysiology Herz‐ und Diabeteszentrum NRW Bad Oeynhausen Germany; ^2^ Department of Cardiology Cardiovascular Research Institute Maastricht (CARIM) Maastricht University Medical Center Maastricht the Netherlands; ^3^ Cardiovascular Division University of Pisa Pisa Italy; ^4^ Department of Biomedical Sciences Faculty of Health and Medical Sciences University of Copenhagen Copenhagen Denmark; ^5^ Centre for Heart Rhythm Disorders University of Adelaide and Royal Adelaide Hospital Adelaide Australia

**Keywords:** atrial fibrillation, high‐density mapping, laboratory analysis, pulmonary vein isolation, workflow optimization

## Abstract

**Background and Aims:**

A novel multielectrode variable loop catheter (VLC) has been introduced for atrial fibrillation (AF) ablation enabling 3D electroanatomic mapping and concomitant pulsed field ablation (PFA). This study sought to investigate the VLC under routine clinical conditions for AF ablation.

**Methods:**

Consecutive patients with symptomatic AF undergoing first‐time AF ablation were prospectively enrolled. All procedures were carried out using the VLC. Electroanatomic mapping pre and post‐ablation was conducted with the VLC and a high‐density multipolar mapping catheter. The general ablation protocol consisted of four ablation pulses per pulmonary vein (PV). All procedures were conducted in conscious sedation.

**Results:**

Forty‐five patients (mean age 66.3 ± 6.1 years, 68.9% paroxysmal AF) were analyzed. Procedure duration was 66.3 ± 13.1 min. Acute pulmonary vein isolation (PVI) was achieved in 45 patients without periprocedural complications. Remapping after the initial 16 ablation pulses revealed sustained electrical conduction to at least one PV in six patients (13.3%). Repeat ablation was conducted and with an average of 7.5 ± 4.5 additional pulses. PV intubation during mapping was achieved in 168/180 PVs with the VLC (93.3%) and in 180/180PVs (100%) with the high‐density mapping catheter (*p* < 0.001). Incomplete PV intubation during mapping did not result in incomplete PVI, as demonstrated by remapping utilizing the high‐density mapping catheter. Adequate correlation between left atrial post‐ablation remapping of low voltage areas and ablated regions was demonstrated in all patients.

**Conclusion:**

PFA‐guided AF ablation using the novel VLC is safe and effective. The integration into a 3D‐electroanatomic mapping system enables adequate mapping during PFA procedures.

**Abbreviations**
ACT: activated clotting time
AF: atrial fibrillation
DOAC: direct oral anticoagulant
IQR: Interquartile range
LA: left atrium
PVI: pulmonary vein isolation
PFA: pulsed‐field ablation
RA: right atrium
SD: standard deviation
TPI: tissue proximity indicator
TEE: transesophageal echocardiography
VLC: variable loop catheter
VKA: vitamin K‐antagonist

## 1 Introduction

Catheter ablation has evolved to the most effective treatment modality for symptomatic atrial fibrillation (AF) [1]. Conventional thermal ablation techniques can be associated with complications such as esophageal injury, pulmonary vein stenosis, and other unintended side‐effects [2]. However, while traditional ablation techniques utilizing thermal energy sources have demonstrated efficacy in pulmonary vein isolation (PVI), they also carry risks of complications and variable procedural success rates [3, 4, 5, 6]. Consequently, there is an increasing interest in alternative ablation technologies that can reduce risks and improve efficacy.

Pulsed‐field ablation (PFA) has emerged as such an alternative, leveraging nonthermal energy to create precise myocardial lesions by electroporation while preserving surrounding anatomical structures [4, 5, 6]. Recently, a novel device utilizing a multi‐electrode variable loop catheter (VLC) has been become commercially available. The VLC is a sizeable decapolar loop catheter system allowing for cardiac mapping and concomitant PFA at selected electrode bipoles [7, 8, 9]. This device has been demonstrated to be effective for the treatment of paroxysmal AF in the pre‐market inspIRE and admIRE trials [10, 11, 12]. Current AF ablation workflows mostly rely on different catheters for intracardiac mapping and ablation. In case of single‐shot ablation, most centers do not use electroanatomic mapping [3]. Additionally, the most commonly used PFA device is not explicitly designed to be incorporated into electroanatomic mapping systems [4]. Potential advantages of the VLC include its utilization for both mapping and ablation which may result in shorter procedures times and decreased necessity for catheter exchanges, potentially decreasing the risk of cardiogenic embolism. Additionally, the VLC can be used with commercially available 8.5F sheaths potentially facilitating less complex access to femoral veins and the left atrium (LA) and improved intracardiac steerability as compared to other PFA ablation devices. There is no further data on utilization of the novel device outside of this pilot studies or under routine clinical conditions. This study aimed to examine the VLC device and its integration into a 3D‐electroanatomic mapping system, under routine clinical conditions within our workflow for PFA‐guided AF ablation.

## 2 Methods

### 2.1 Patient Population

Consecutive patients suffering from symptomatic paroxysmal or persistent AF undergoing index AF ablation with the VLC (Varipulse, Biosense Webster, Irvine, CA, USA) between April 2024 and December 2024 at our center were prospectively enrolled in our institutional ablation registry for all‐comers catheter ablation at our centre (ethical review board file number 2019‐563). Patients with longstanding‐persistent AF and large LA dimensions (LA diameter measured by transthoracic echocardiography >55 mm in parasternal long‐axis or LA volume index >50 mL/m^2^) were excluded from receiving ablation with the VLC device. The study was approved by the local ethics board and complied with the declaration of Helsinki.

### 2.2 Ablation Procedure Workflow

Ablations were carried out by three different experienced operators who performed more than 500 previous AF ablation procedures. Transesophageal echocardiography (TEE) was conducted in patients lacking continuous oral anticoagulation prior to the procedure and at risk for thromboembolism, or in those with a history of stroke or thromboembolic events, to rule out intracardiac thrombi. All procedures were performed on uninterrupted vitamin K‐antagonist (VKA) therapy (target INR 2.0–3.0). Direct oral anticoagulant (DOAC) therapy was withheld on the day of the procedure and reinitiated 6 h after the procedure.

Catheter ablation was performed under deep intravenous sedation with boli of midazolam and sufentanil and continuous propofol infusion [13]. Drug administration and monitoring was carried out by qualified nurses and under supervision of the treating physicians. Heparin was administered targeting an activated clotting time (ACT) >300 s; the first dose of heparin was administered after gaining femoral vein access. The VLC was introduced into the LA only when ACT levels >300 s were achieved. All patients received preprocedural imaging by computed tomography or cardiac magnetic resonance imaging to evaluate left atrial and pulmonary vein anatomy and to guide the ablation procedure. Ablation was performed with the use of an electroanatomic mapping system (CARTO 3 V8, Biosense Webster). In addition, an 8.5F steerable sheath (ViziGo, Biosense Webster) allowing for visualization inside the electroanatomic mapping system was used [14, 15].

Femoral access was gained by placing a 7F and an 8F sheath in the right femoral vein under sonographic guidance. After placement of a decapolar diagnostic catheter inside the coronary sinus, electroanatomic mapping of the right atrium (RA) utilizing a high‐density mapping catheter (Pentaray, Biosense Webster) was performed to create a matrix for visualization of the steerable sheath [14, 15]. Afterwards LA access was obtained by a single transseptal puncture with the steerable sheath and a BRK‐1 needle (St. Jude Medical, St Paul, MN, USA), followed by selective angiographies of all PVs and high density electroanatomic mapping of the LA and PVs. Mapping was conducted in order to determine left atrial geometry to facilitate PVI and to identify low voltage areas in the left atrium, defined as regions with a bipolar voltage of less than 0.5 mV (during mapping in sinus rhythm). Mapping with the VLC was conducted with a closed loop configuration to enhance maneuverability. The tissue proximity indicator (TPI) of the VLC device was trained during mapping. In case of suspected VLC movement during energy delivery the catheter position was verified by inspection of simultaneous movement of the coronary sinus catheter (in case of false catheter position display by the mapping system during energy delivery) and with fluoroscopy.

**FIGURE 1:**
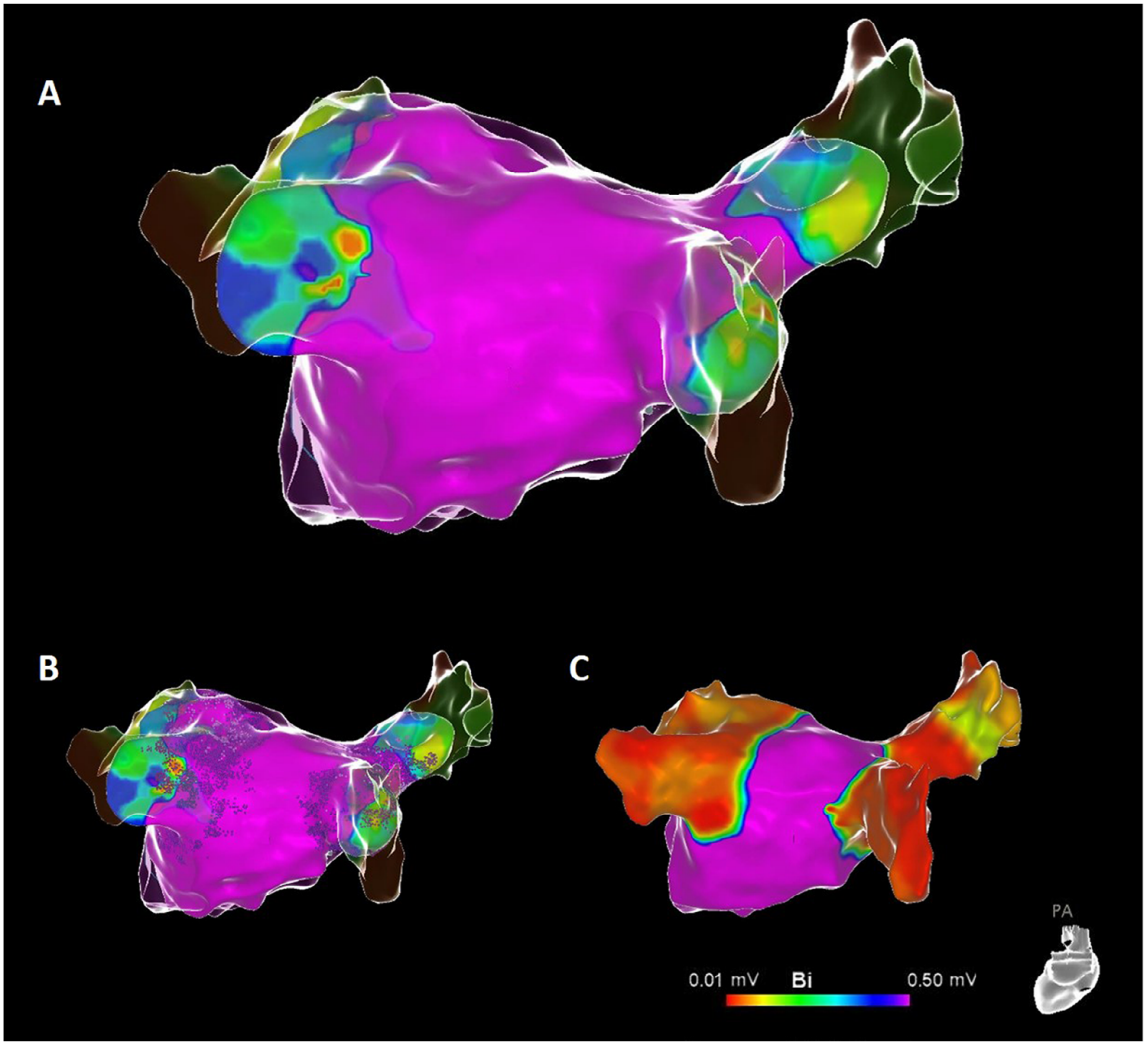
Electroanatomical mapping utilizing high‐density multielectrode catheters and the multi‐eletrode variable loop catheter. Overlay of electroanatomical maps with a high‐density multielectrode catheter (glass mode) and the VLC device (color‐coded map) (A). All PVs were successfully engaged with the VLC device for mapping. Localization of ablation spots is visualized by grid tags (B). Post‐ablation remapping with a multielectrode catheter demonstrates good correlation between low‐voltage areas and ablation tags (C). [Colour figure can be viewed at wileyonlinelibrary.com]

**FIGURE 2:**
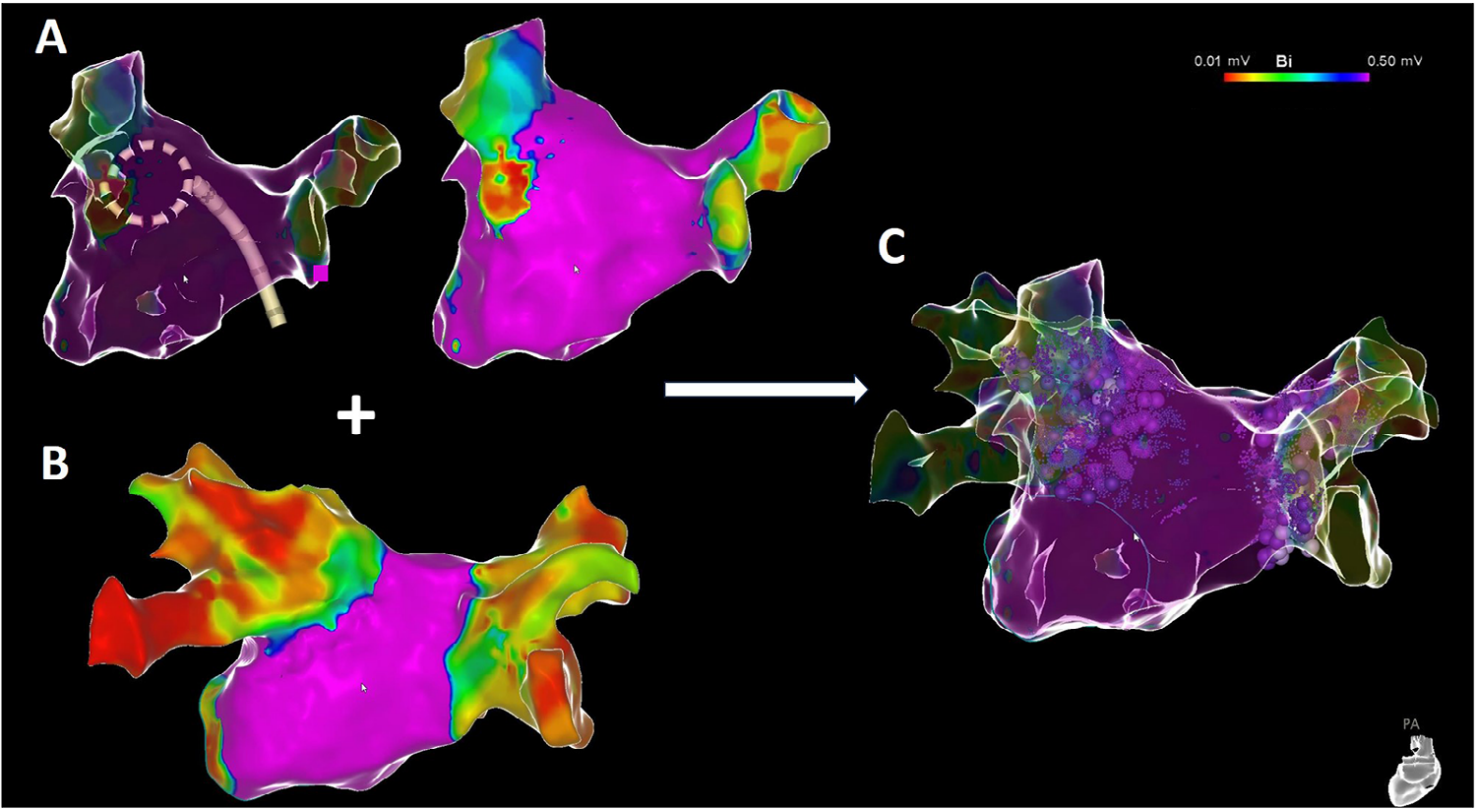
Correlation of electroanatomic mapping and ablation localization. Representative electroanatomical maps with the VLC device (A) and a multielectrode catheter (B) as well as an overlay of both maps with integrated ablation tags (C). The map shown in (A) depicts a case in which intubation of the LIPV was not successful with the VLC device. Nevertheless, remapping after ablation with a high‐density mapping catheter (B) demonstrated successful ablation in this case. [Colour figure can be viewed at wileyonlinelibrary.com]

Procedural endpoint was PVI with proof of entrance and exit block in all PVs. The VLC was introduced into the LA via commercially available 8.5F sheaths. The ablation protocol followed the recommendations with two ostial and two antral PFA ablations for each PV, resulting in 16 ablations for PVI per patient before repeat mapping was attempted [10, 11, 12]. Each ablation consisted of a delivery of three trains of bipolar energy delivery with 1800 V for 250 ms with a pause of 10 s between each application. Delivery of PFA was only possible without spatial electrode overlapping; in cases with overlapping the ablation system allows for selected inactivation of electrode pairs to enable energy delivery. Ablation was guided by impedance‐based tissue contact visualization at the electrodes of the VLC device. Localizations of energy delivery were manually tagged inside the electroanatomic map to visualize ablation lesion localizations (Figures 1B and 2C). Remapping to confirm PVI and to visualize the effect of ablation was performed in all patients and additional PFA applications were administered to accomplish persistent PVI in the event of sustained electrical LA to PV conduction following initial ablation. In patients with bipolar low voltage areas in the LA, further ablations were carried out at the operators' discretion. In case of low voltage areas at the posterior wall, additional ablation was performed aiming at creation of a posterior “box lesion” with entry and exit block of the entire LA posterior wall area.

### 2.3 Post‐Procedural Care

After sheath removal a figure of eight suture was applied and compression of the puncture side was applied by a bandage for 6 h and pericardial effusion was excluded by transthoracic echocardiography. Patients received proton‐pump inhibitors for 4 weeks. Anti‐arrhythmic drug therapy was prescribed on an individual basis for each patient. Patient's discharge followed the day after ablation.

### 2.4 Statistical Analysis and Assessment of Electroanatomic Mapping Effectiveness

**FIGURE 3:**
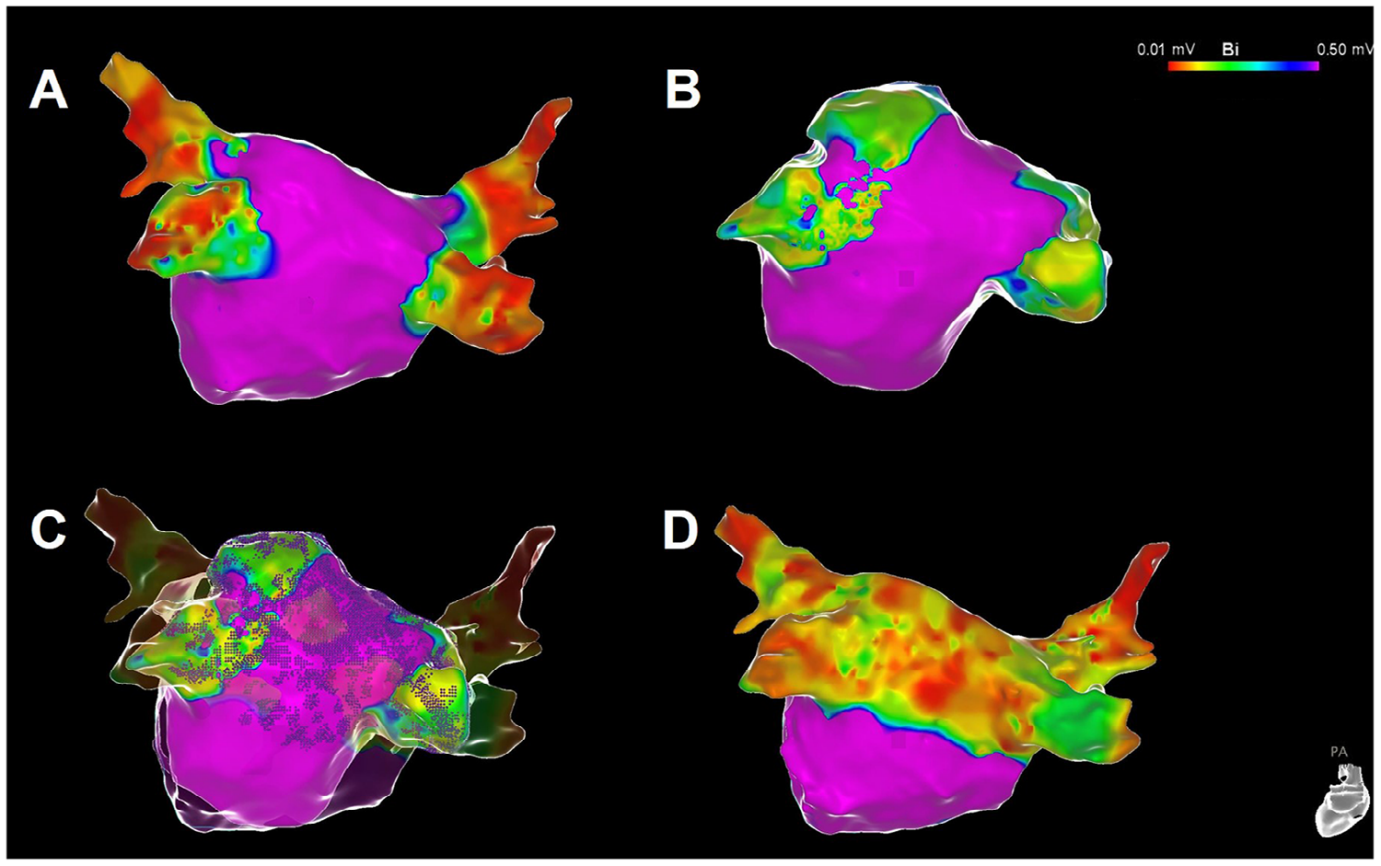
Left atrial substrate modification with posterior wall ablation using the multielectrode variable loop catheter. Electroanatomical maps with multielectrode catheter (A), VLC device (B), overlay maps with integrated ablation tags (C), and post‐ablation remap demonstrating homogenous low‐voltage (C) in a patient receiving PVI and box isolation. Notably, complete intubation of the RSPV was not possible with the VLC device. [Colour figure can be viewed at wileyonlinelibrary.com]

Continuous variables were expressed as mean ± standard deviation (SD) for normal distributions or as median/interquartile range (IQR) for non‐normal distributions or categorical data. Categorial variables were displayed as counts (%). Comparison of contingency tables was performed with a Fisher's exact test, a *p* value <0.05 was considered as statistically significant. Electroanatomic mapping was performed using a multielectrode catheter as well as the VLC device to allow for comparison of mapping (Figures 1, 2, 3). Electroanatomic mapping was conducted at least once before to and following PVI with both comparators. Complete PV intubation was defined as successful placement of the utilized mapping device in a true ostial and an antral position. The congruent correlation of low voltage areas and ablation tags was assessed by two operators, and it was considered sufficient if both investigators determined that the correlation was adequate based on the analysis of the overlay of mapping and ablation tags. The area of post‐ablation low voltage was compared to the area which was covered by ablation tags and grids; measurement of area (in cm^2^) and area perimeter (in cm) was performed inside the CARTO software.

## 3 Results

### 3.1 Patient Cohort

The study cohort consisted of 45 patients (mean age 66.3 ± 6.1 years, 19 females (42.2%)) with 31 patients (68.9%) suffering from paroxysmal AF and 14 patients (31.1%) from persistent AF. All patients had normal left ventricular ejection fraction. Baseline parameters are depicted in Table 1.

**TABLE 1:** Patients baseline characteristics.

Parameter	Value
Patients, *n*	45
Female, *n* (%)	19 (42.2)
Age (years)	66.3 ± 6.1
BMI (kg/m^2^)	27.9 ± 6.2
LVEF (%)	54.2 ± 1.9
Paroxysmal AF, *n* (%)	31 (68.9)
Persistent AF, *n* (%)	14 (31.1)
Arterial hypertension, *n* (%)	30 (66.7)
Diabetes mellitus, *n* (%)	28 (62.2)
History of stroke/TIA, *n* (%)	3 (6.7)
CHA2DS2‐VA score, median (IQR)	2 (2;4)
Anticoagulation pre ablation, *n* (%)	37 (82.2)
DOAC, *n* (%)	37 (82.2)
Antiarrhythmic drug therapy pre ablation, n (%)	15 (33.3)
Class I, *n* (%)	8 (17.8)
Class III, *n* (%)	7 (15.6)

*Note*: Data are presented as *n* (%) or mean ± standard deviation.

Abbreviations: AF = atrial fibrillation, BMI = body mass index, DOAC = direct oral anticoagulant, LVEF = left ventricular ejection fraction, TIA = transitoric ischemic attack.

### 3.2 Procedural Data

Procedural data are shown in Table 2. There were 27 patients (60.0%) in sinus rhythm at the start of procedure while the remaining patients (18, 40.0%) were in AF. All procedures were carried out in intravenous sedation and no patient needed conversion to general anesthesia. Procedure duration (femoral vein puncture to sheath removal) was 66.3 ± 13.1 min (ranging from 43–113 min) with mean fluoroscopy times of 3.7 ± 1.8 min. Acute PVI at the end of the procedures was achieved in all 45 patients (100%; 180/180 PVs). This was also the case in two patients with a common left PV ostium in whom all left PV branches were selectively targeted by ablation.

**TABLE 2:** Procedural data.

Parameter	Value
Intravenous sedation, *n* patients	45 (100.0)
General anesthesia, *n* patients	0 (0.0)
Number of operators	3
Mean procedural duration (min)	66.3 ± 13.1
Mean fluoroscopy time (min)	3.7 ± 1.8
Median fluoroscopy dosage (γ Gy × m^2^)	256 (IQR 96.6, 357.4)
Ablation strategies	
Attempted PVI, *n* patients	45 (100.0)
Complete PVI (after first 16 ablations), *n* patients	39 (86.7)
Isolated PVs (after first 16 ablations), *n* PVs	167/180
Preserved electrical conduction (at least 1 PV, remapping after PFA), *n* patients	6 (13.3)
Complete PVI (end of procedure), *n* patients	45 (100.0)
Isolated PVs (end of procedure), *n* PVs	180/180
Additional ablation to PVI, *n* patients	8 (17.8)
Box lesion, *n* patients	5 (11.1)
Complete box isolation, *n* patients	5 (11.1)
Anterior substrate modification, *n* patients	3 (6.7)
PFA ablations (total), *n*	19.4 ± 8.5
PFA ablations (PVI), *n*	18.5 ± 3.7
PFA ablations (touch‐up PVI, 6 cases), *n*	7.5 ± 4.5
PFA ablations (additional ablation), *n*	5.1 ± 1.4

*Note*: Data are presented as *n* (%), mean ± SD or median with interquartile range.

Abbreviations: IQR = interquartile, PFA = pulsed‐field ablation, PV = pulmonary vein, PVI = pulmonary vein isolation.

Remapping utilizing the VLC and the multipolar mapping catheter after the initial 16 ablation pulses revealed sustained electrical conduction to 14/180 PVs (7.8% of PVs) in six patients (13.3% of patients, 3/31 with paroxysmal AF and 3/14 with persistent AF, *p* = 0.35). In one patient all four PVs received additional ablations due to sustained electrical conduction, in two patients the left superior PV (LSPV) and left inferior PV (LIPV) needed additional ablations, in one patient the LSPV, LIPV, and RSPV needed additional ablations and in two patients, additional ablations at the right inferior PV (RIPV) were performed. Repeat ablation to attain PVI was conducted and succeeded in all these patients, with an average of 7.5 ± 4.5 and a maximum of 16 additional (touch‐up) ablations.

Additional ablation beyond PVI was performed in eight patients (17.8%) who suffered from persistent AF. No additional ablation was performed in patients with paroxysmal AF. Based on the presence of bipolar LA low voltage, five patients (11.1%) with persistent AF received a posterior box lesion. In addition, three patients (6.7%) who suffered from persistent AF received additional ablation at the anterior/septal wall, due to unexpected occurrence of LA macro‐reentrant tachycardia in addition to the presence of low voltage areas. Left atrial posterior box isolation resulted in complete signal loss as well as exit block demonstrated by pacing from the ablation device and/or mapping catheter in all five patients. A mean of 19.4 ± 8.5 ablations were delivered in the total patient cohort, with a mean of 18.5 ± 3.7 ablations (range 16–32) aiming at PVI and 5.1 ± 1.4 ablations (range 4–11) for additional ablations. No periprocedural complications were observed and all patients were discharged the day after ablation.

### 3.3 Analysis of Mapping Performance

Figures 1, 2, 3 show representative examples of mapping attempts as compared to the standard high‐density multi‐electrode mapping catheter and the ablation device. The VLC device effectively engaged 168/180 PVs (93.3%; unsuccessful PV intubation: 2xLSPV, 6xLIPV, 1xright superior PV [RSPV], and 3xRIPV), while the high‐density mapping catheter successfully engaged all PVs (Fisher exact *p* value for comparison of mapping catheter and VLC < 0.001) (Table 3). In case of failed PV intubation, the ablation device was successfully placed at the PV antrum and PFA applications were delivered in two different loop sizes. Following this approach, incomplete PV intubation did not result in ineffective PVI, as demonstrated by remapping using the high‐density mapping catheter. Incomplete PV intubation was also not related to the necessity of touch‐up ablations since all of these PVs were isolated after the initial 16 ablations, as demonstrated by remapping using the high‐density mapping catheter. Adequate correlation between LA post‐ablation remapping of low voltage areas and ablated regions was demonstrated in all patients, as judged by the operators (Table 3). The mean area of post‐ablation low voltage areal for left and right PVs was comparable to the covered area of ablation tags and grids (45.1 ± 5.2 cm^2^ vs. 44.7 ± 5.0 cm^2^ for left PVs, *p* = 0.67; 58.3 ± 11.8 cm^2^ vs. 58.4 ± 11.8 cm^2^ for right PVs, *p* = 0.98) (Table 3). The mean perimeter of post‐ablation low voltage areas and the mean perimeters of areas covered by ablation tags/grids did also not differ for left and right PVs (13.1 ± 1.6 cm vs. 13.0 ± 1.5 cm for left PVs, *p* = 0.88; 16.1 ± 3.5 cm vs. 17.0 ± 3.6 cm for right PVs, *p* = 0.83) (Table 3).

**TABLE 3:** Electroanatomic mapping data.

Parameter	Mapping catheter	VLC	*p* value
Successful complete PV intubation during mapping, *n*	180/180 PVs (100%)	168/180 PVs (93.3%)	<0.001
Congruent correlation of ablation tags with post‐ablation low voltage areas, *n* (operator judgement)	45/45 patients (100%)	45/45 patients (100%)	1.0
Mean area of post‐ablation low voltage or area covered with ablation tags/grids (left PVs)	45.1 ± 5.2	44.7 ± 5.0	0.67
Mean area of post‐ablation low voltage or area covered with ablation tags/grids (right PVs)	58.3 ± 11.8	58.4 ± 11.8	0.98
Mean perimeter of area of post‐ablation low voltage or area covered with ablation tags/grids (left PVs)	13.1 ± 1.6	13.0 ± 1.5	0.88
Mean perimeter of area of post‐ablation low voltage or area covered with ablation tags/grids (right PVs)	16.1 ± 3.5	17.0 ± 3.6	0.83

Abbreviation: PV = pulmonary vein.

## 4 Discussion

This single‐center real‐world observational analysis on utilization of a novel PFA device dedicated to AF ablation has the following major findings: (1) PFA‐guided AF ablation using the novel VLC was safe and effective; (2) the device's integration into a 3D‐electroanatomic mapping system was useful, providing adequate mapping during the procedures; and (3) the VLC allows for a single catheter AF (mapping and) ablation workflow under routine clinical conditions.

### 4.1 The Novel VLC Device and Current Technology for Pulsed Field Ablation in Atrial Fibrillation Ablation

PFA has emerged as a rapidly growing technology in cardiac electrophysiology, providing a save and effective treatment for AF. To date, over 100.000 AF ablation procedures have been performed under routine clinical conditions using another commercially available PFA device. This system utilizes a 13F inner‐size sheath and an over‐the‐wire technique. In a randomized controlled trial conducted on patients with paroxysmal AF, this PFA device proved to be non‐inferior in terms of freedom from arrhythmia recurrence when compared to thermal energy sources [16]. This device has also been shown to be highly time‐effective in terms of time‐efficient ablation, too. Typically, procedural durations are reported to be around 60 min [17]. The single‐center 5S study reported the streamlining of a procedural protocol for AF ablation using PFA, which resulted in brief procedure durations of only 39 ± 14 min on average [18]. It is important to mention that in over 86% of the patients in this study, no 3D electroanatomic mapping or general anesthesia was employed, which may have contributed to the relative short procedure durations [18].

We report on procedure durations that are less than 70 min using the VLC. All procedures in our study were conducted with the utilization of a 3D electroanatomic mapping system. Additionally, we were able to perform VLC‐guided PFA without the necessity of general anesthesia. Our results regarding procedural parameters are consistent with the results of the inspIRE and admIRE trials that were previously published [10, 11, 12]. Notably, we did not observe any serious adverse events in our patient cohort, consistent with the initial inspire trial [10]. In the admIRE trial the rate of adverse events was 2.9%, including 1.1% of patients with cardiac tamponade and 1.1% of patients suffering from cerebrovascular events or transient ischemic attacks [12]. We were able to conducting all LVC‐guided ablation procedures with conscious sedation alone, which is In contrast to the inspIRE study in which about 70% of patients underwent ablation under general anesthesia [10]. This finding has substantial effects on the system's broad applicability, particularly in centers with restricted access to general anesthesia for AF ablation procedures.

### 4.2 Features of the Novel Ablation Device

**FIGURE 4:**
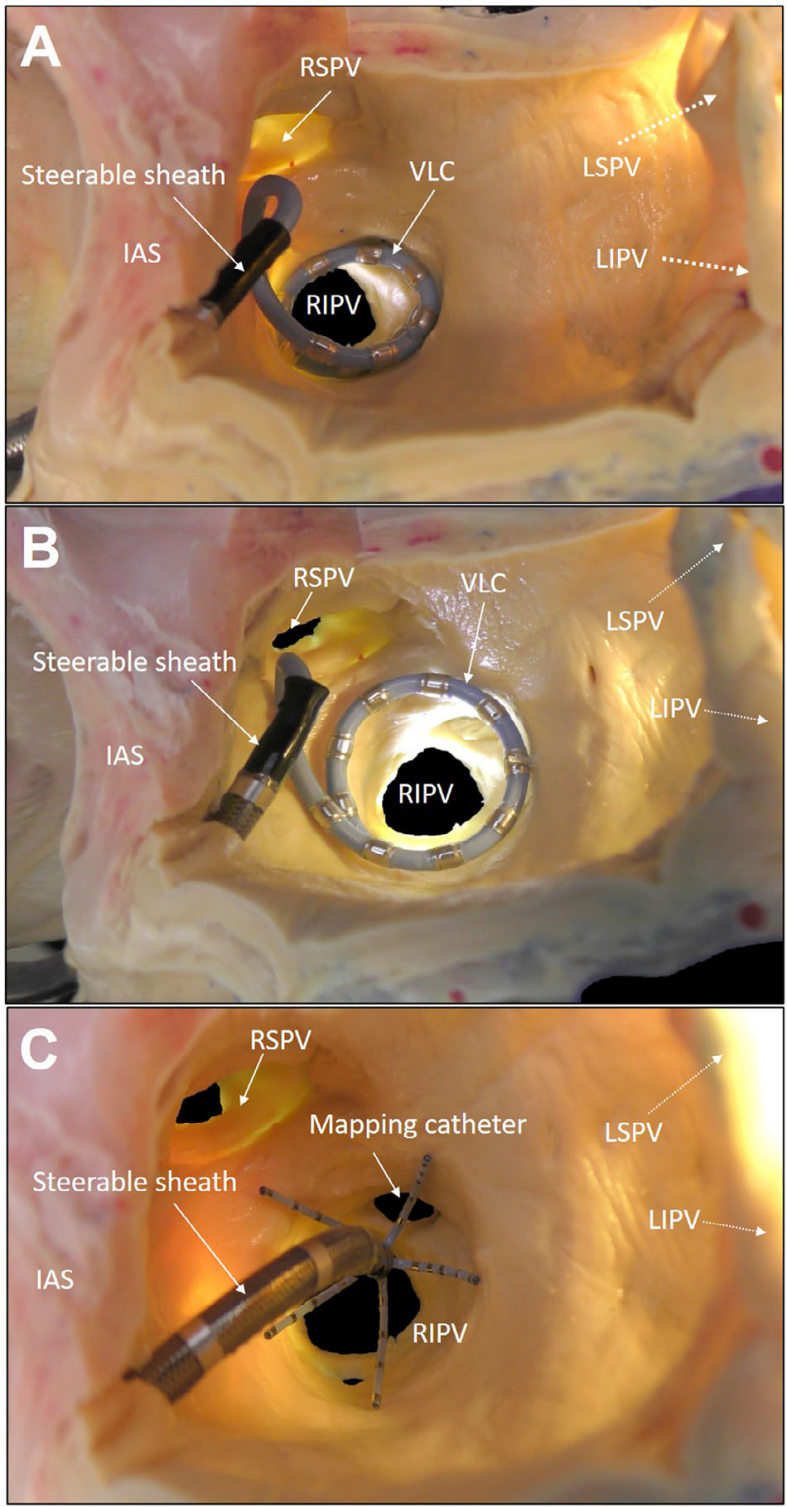
Anatomic specimen of the left atrium demonstrating catheter placement. Representative images showing catheter placement inside the LA of an anatomic specimen. (A) Placement of the VLC in an ostial position at the right inferior pulmonary vein (RIPV), utilizing the contraction knob to adjust the device's diameter. (B) Placement of the VLC in an antral position after retraction from the ostial location and increase in the device diameter. The placement of a dedicated mapping catheter at the RIPV ostium is also shown. IAS = intraatrial septum, LIPV = left inferior pulmonary vein, LSPV = left superior pulmonary vein, RIVP = right inferior pulmonary vein, RSPV = right superior pulmonary vein, VLC = variable loop catheter. [Colour figure can be viewed at wileyonlinelibrary.com]

This novel ablation system is fully integrated into an electroanatomic mapping system, enabling the simultaneous execution of ablation and mapping. In contrast to other commercially available single‐shot PFA devices, it can be introduced using standard 8.5F sheaths, which simplifies the adoption process for routine users of conventional ablation systems, such as radiofrequency ablation. In comparison to other PFA devices, the maneuverability may be enhanced by the absence of over‐the‐wire steering (Figure 4). The device's variable size enables it to be adjusted to appropriately engage different types of variant PV anatomy. A larger number of patients must be examined to accurately assess the efficacy of PFA with the new device in patients with normal and variant PV anatomy. The device's integration into the 3D mapping system is another advantage, as it enables the precise labeling of every PFA application and the disabling of tagging for deactivated electrodes in the event of electrode overlapping. This feature resulted in a high degree of precision in the overlap of ablation tags and low voltage areas in our patient cohort.

At present, there is no data with regards to long‐term clinical efficacy to support the use or non‐use of 3D electroanatomic mapping for PFA‐based AF ablation. In the admIRE and inspIRE trials, the rates of successful PVI were mostly assessed by remapping with the VLC device [19]. In the inspIRE study 86% of patients were mapped and ablated with a single catheter without any catheter exchange [10]. Previously published data show that about 9%–15% of patients who undergo PFA with another commercially available over‐the‐wire ablation device have persistent LA‐PV conduction after the first ablation when remapping with a 3D mapping system and a dedicated mapping catheter is used [18, 20]. In contrast to a pure fluoroscopic approach, Badertscher et al. did not observe a prolonged arrhythmia‐free survival following PFA‐based PVI when 3D mapping was employed [19]. However, it is crucial to note that remapping was conducted in this report after PFA, and sustained LA to PV conduction was observed in 9% of cases followed by immediate repeat ablation of these PVs [20]. This finding is consistent with our results, which indicated that 12% of patients had incomplete PVI following initial ablation and remapping. The clinical implications of this finding are not yet known. In contrast to the above mentioned admIRE and inspIRE trials, which mainly focused on procedural safety and clinical effectiveness, we investigated the specific ability of the VLC for mapping in comparison to a high‐density mapping device. Our data shows, that mapping and ablation is feasible using only a single AF ablation device without the need for an additional dedicated mapping catheter. Other approaches may incorporate additional imaging techniques such as intracardiac echocardiography [21]. In this pilot study, we utilized both the high‐density mapping catheter and the LVC in all patients and an adequate correlation between LA post‐ablation remapping of low voltage areas and ablated regions was demonstrated. Moreover, the VLC successfully engaged a significant number of PVs, and the PFA applications resulted in the complete isolation of all targeted veins, even in cases where complete intubation was not feasible due to anatomical considerations. In this study, the single use of a single catheter approach for mapping and ablation catheter was not directly investigated. In terms of PV intubation, we discovered that the dedicated mapping catheter was preferable to the LVC device. Nevertheless, complete PVI was also demonstrated in PVs in which the LVC device could not be positioned in a true ostial position, while conventional catheter configurations were used. Further research is required to determine whether the novel PFA workflow for AF, which does not require an additional dedicated high‐density mapping catheter, would result in comparable procedural success when used for mapping and concomitant ablation.

### 4.3 Limitations

This prospective, non‐randomized study is limited in sample size, despite being the largest of its kind to date, and has been conducted at a single center. We did not evaluate the feasibility of mapping and ablation alone on the basis of the information collected with the LVC device within the electroanatomic mapping system, as a multipolar catheter was utilized in all patients. Additionally, we performed intraprocedural PV angiographies and preprocedural imaging on all patients. The current data provides a detailed description of the ablation workflow without any follow‐up to evaluate the clinical efficacy of this innovative ablation approach in the mid‐ and long‐term. The accuracy of the mapping was partially analyzed using operator interpretation, which could be biased by human error.

## 5 Conclusion

PFA‐guided PVI using the novel VLC catheter was safe and effective. The device's integration into a 3D‐electroanatomic mapping system was useful, providing adequate mapping during the procedures.

## Conflicts of Interest

TF received advisory fees from Boston Scientific and Adagio Medical. PS is member of the advisory board of Abbott, Biosense Webster, Boston Scientific and Medtronic. C. Sohns received lecture fees and is a consultant for Biosense Webster, Boston Scientific and Medtronic. All remaining authors have declared no conflicts of interest.

## Declaration of Generative AI and AI‐Assisted Technologies in the Writing Process

During the preparation of this work the author(s) used QuillBot to check for grammar and spelling. After using this tool/service, the author(s) reviewed and edited the content as needed and take(s) full responsibility for the content of the publication.

## Data Availability

The data that support the findings of this study are available on request from the corresponding author. The data are not publicly available due to privacy or ethical restrictions.
